# Preoperative Transcranial Doppler Findings and Postoperative Delirium After Cardiac Surgery in Elderly Patients: A Prospective Observational Study

**DOI:** 10.3390/life16061026

**Published:** 2026-06-19

**Authors:** Astrid Bergmann, Yurii Ruzhyn, Jan Wiesemann, Nikolai Hulde, Janis Fliegenschmidt, Alexander Krannich, Vera von Dossow

**Affiliations:** 1 Heart- and Diabetes-Center Bad Oeynhausen, Ruhr-University Bochum, 32545 Bad Oeynhausen, Germanyvvondossow@hdz-nrw.de (V.v.D.); 2 Heart- and Diabetes-Center Bad Oeynhausen, Medical Center East Westphalia-Lippe, University Bielefeld, 32545 Bad Oeynhausen, Germany; 3 Knappschaft Kliniken, Ruhr-University Bochum, 44892 Bochum, Germany; 4 BioStats GmbH, 14641 Nauen, Germany

**Keywords:** postoperative delirium, cardiac surgery, transcranial Doppler, cardiopulmonary bypass, cerebrovascular reserve, cerebral autoregulation

## Abstract

Postoperative delirium (POD) is a common neurocognitive complication after cardiac surgery in elderly patients and is associated with adverse clinical outcomes. Impaired cerebral autoregulation and reduced cerebrovascular reserve may contribute to POD development. Automated transcranial Doppler sonography (TCD) enables non-invasive assessment of intracranial hemodynamics and may provide additional information for perioperative risk assessment. In this prospective single-center observational study, 108 patients aged >70 years scheduled for elective cardiac surgery with cardiopulmonary bypass were enrolled. Patients who had pre-existing neurological disease, had a pathological carotid Doppler ultrasound, underwent emergency surgery, or were unable to undergo delirium screening were excluded. Preoperative bilateral TCD of the middle cerebral arteries was performed using an automated WAKIe R3 system. POD was assessed on postoperative days 1–3 using the CAM-ICU. The primary endpoint was the occurrence of POD. Twenty-one patients were excluded, leaving 87 patients for analysis. POD occurred in 14 patients (16%). All patients who developed POD had pathological preoperative TCD findings, whereas no POD occurred among patients with normal TCD examinations. Overall, 82 patients (94%) demonstrated pathological intracranial hemodynamic findings despite normal carotid Doppler ultrasound. In multivariable Firth logistic regression adjusted for age and sex, pathological TCD findings remained associated with POD; however, interpretation was limited by the small number of outcome events and quasi-complete separation. In elderly patients undergoing cardiac surgery with cardiopulmonary bypass, pathological preoperative TCD findings were frequently observed and may be associated with an increased risk of postoperative delirium. The marked discrepancy between normal carotid ultrasound and abnormal intracranial hemodynamics suggests that TCD may provide complementary information regarding cerebrovascular function. Given the limited sample size and event rate, these findings should be considered exploratory and require confirmation in larger multicenter studies.

## 1. Introduction

Postoperative delirium is a neurocognitive disorder characterized by acute changes in cognition and awareness and fluctuating disturbances in attention. It occurs frequently among elderly patients, with the incidence ranging from 28 to 83% [1], especially after high-risk surgery [2,3]. A high postoperative delirium rate after cardiac surgery using a heart–lung machine has been described, ranging from 4 to 73% [4,5]. It has also been demonstrated that this has a detrimental impact on patients by prolonging the length of stay in the intensive care unit and the overall length of stay in hospital by 2–5 days and increasing the risk of death in long-term follow-up by approximately 2–4 fold [6,7,8,9]. Such patients will most probably not regain the quality of life experienced prior to surgery [10], and they are likely to be discharged to rehabilitation or nursing facilities rather than home [11]. Postoperative delirium thus represents a significant socioeconomic burden [12,13].

It is important to understand the pathophysiology of delirium and to identify potential risk factors to provide optimal care to the patient and to prevent delirium or at least reduce its impact [14]. Among the many risk factors associated with the development of delirium, previous studies have consistently identified the duration and severity of intraoperative hypotension as significant contributors [15]. However, prospective randomized trials using different thresholds for blood pressure management have shown inconsistent results [16,17,18].

Impaired cerebral autoregulation and a diminished cerebral flow reserve have likewise been recognized as potential risk factors for the development of postoperative delirium [19]. Therefore, the question arises as to whether, in addition to blood pressure, altered cerebral blood flow also plays a role, or even a more important one. Cerebral blood flow is not routinely recorded pre- or intraoperatively. Still, it might provide valuable information as a monitoring parameter during surgery.

Transcranial Doppler sonography is a non-invasive procedure to directly measure cerebral blood flow velocity. Thus, it provides significant information on cerebral hemodynamics. It has been demonstrated that parameters such as the pulsatility index, the mean flow velocity of the median cerebral artery, and the breath-hold index can be associated with adverse neurological outcomes [20,21,22]. Its use for intraoperative monitoring has also demonstrated advantages in maintaining adequate blood flow [23].

Transcranial Doppler sonography has so far been used to a limited extent in routine clinical practice, as there was initially no automated, bilateral device at hand. This task always required an additional examiner to operate an ultrasound scanner [24]. The development of self-adjusting measurement devices has helped overcome this limitation, enabling transcranial Doppler to be used as a practical monitoring tool for the straightforward assessment of cerebral blood flow. Still, there have been no randomized controlled trials to assess the clinical value of its use for preoperative risk assessment in cardiac surgery. Therefore, it remains unclear whether a direct evaluation of the cerebral blood flow allows for more sophisticated risk stratification und thus improves patients’ neurological outcomes.

At the authors’ hospital, more than 6000 patients are annually submitted to cardiac surgery. As in most cardiac centers, patients above 70 years of age are most common, i.e., patients with an increased risk of developing postoperative delirium [14]. The aim of this prospective, single-center observational study was to evaluate whether automated, non-invasive bilateral cerebral blood flow measurement, used as a risk stratification tool, influences neurological outcomes in elderly patients undergoing elective cardiac surgery under cardiopulmonary bypass.

## 2. Materials and Methods

### 2.1. Patients

In total, 108 patients scheduled for elective cardiac surgery with cardiopulmonary bypass were enrolled in this prospective observational study. The study period was from January 2024 until December 2024. All patients were over 70 years of age, and our exclusion criteria were pre-existing neurological or psychiatric disorders that could interfere with cerebral hemodynamics or postoperative cognitive assessment, including dementia, previous stroke or transient ischemic attack, Parkinson’s disease, epilepsy, multiple sclerosis, and other neurodegenerative diseases, as well as major psychiatric disorders such as schizophrenia or severe depression. Patients with a pathological preoperative carotid Doppler ultrasound were also excluded. Further exclusion criteria included emergencies requiring surgical intervention, as well as an inability or refusal to undergo delirium screening. The surgical procedures ranged from coronary artery bypass graft to aortic, mitral, and tricuspid valve replacement or repair, or a combination of bypass and valve surgery. Demographical and clinical data were collected and saved in the patients’ electronic records; the study data were stored separately. The ethics committee of the Ruhr-University Bochum, Germany, approved the protocol for this study (OWL, AZ 2023-1081) on 14 July 2023. All patients gave their written informed consent according to the Declaration of Helsinki.

### 2.2. Transcranial Doppler Ultrasound

The patients underwent transcranial doppler ultrasound of the middle cerebral artery (MCA) preoperatively using WAKIe R3 TCD (Atys medical, Soucieu en Jarrest, France). According to the manufacturer’s instructions [25], patients were positioned on a chair to provide easy access to the regions of interest, which were the temples on either side. After coupling gel was spread onto the patient’s skin, the spectacle frame was put on the patient such that there was no hair between the skin and the probe. The headset was adjusted using Velcro and the headband. The optimal temporal acoustic window was identified before fixation of the headset, and signal quality was verified via visual inspection of the Doppler waveform and signal intensity provided by the device software. Measurements were accepted only when stable bilateral recordings of the middle cerebral arteries were obtained. In cases of insufficient signal quality or an inability to identify an adequate temporal bone window, patients were excluded from further analysis. The automated self-adjusting probe positioning and signal optimization functions of the WAKIe R3 system were used throughout the study to improve measurement consistency and reduce operator dependency.

All transcranial Doppler examinations were performed by investigators trained in cerebrovascular ultrasound and familiar with the use of the WAKIe R3 system. Prior to study initiation, the operators underwent device-specific training according to the manufacturer’s recommendations and performed supervised examinations to ensure consistent acquisition of measurements. To minimize interoperator variability, all examinations were conducted according to a standardized study protocol. The mean duration of the transcranial Doppler examination was approximately twenty minutes per patient.

The results yielded by the WAKIe R3 were the systolic peak velocity (SPV), end diastolic velocity (EDV), mean flow velocity (MFV), and pulsatility index (PI; SPV-EDV/MFV) for the right and left MCA separately.

To assess cerebral autoregulation, the ipsilateral carotid artery was carefully compressed mechanically for five seconds, and the aforementioned velocities were recorded immediately afterwards. The transient hyperemic response ratio (THRR) was calculated as the increase in MFV divided by its baseline after the mechanical compression was released, reflecting the autoregulation index [26].

To assess the cerebral reserve capacity, the breath-hold index (BHI) was calculated as follows: Patients were encouraged to hold their breath for 30 s, and the velocities were again recorded. The BHI was the increase in MFV divided by the time of the breath-hold in seconds [20].

The TCD was considered to be pathological in case there was a difference of more than 25% in the SPV between both sides, hinting at a perfusion deficit, and/or in case carotid compression resulted in a more than 50% decline in the ipsilateral MFV and/or a THRR below 1.1, which hinted at impaired cerebral autoregulation, and/or a BHI below 0.69, indicating impaired cerebral reserve capacity [27].

### 2.3. Anesthetic Management

In the induction room, patients were placed on a heating blanket (Twinwarm BB, Moeck & Moeck GmbH, Hamburg, Germany) and equipped with the standard monitoring consisting of a 5-lead-electrocardiogram (Philips IntelliVue patient monitoring system, Philips Medizin Systeme GmbH, Böblingen, Germany), oxygen saturation clip (M1191B, Philips Medizin Systeme GmbH, Böblingen, Germany) on the right index finger, arterial cannula (Arrow arterial catheterization set, Teleflex Incorporated, Wayne, PA, USA) for continuous measurement of the arterial pressure in the left radial artery, and peripheral venous access (Vasofix Safety, B.Braun Melsungen AG, Melsungen, Germany) in the lower arm, either on the right or on the left side, for the application of volume, induction medication, and continuous anesthetic drugs throughout the process. After generous preoxygenation with FiO_2_ 1, general anesthesia was induced intravenously with Sufentanil (0.05–0.1 µg/kg, Janssen-Cilag GmbH, Neuss, Germany), Etomidate (0.2–0.3 mg/kg, B. Braun Melsungen AG, Melsungen, Germany), and Rocuronium (0.6–0.9 mg/kg, Organon / MSD, OSS, Netherlands). After the onset of muscle relaxation, an endotracheal tube was inserted under laryngoscopy. The tube was secured with adhesive tapes. General anesthesia was maintained with the continuous application of Propofol (3–4 mg/kg/h, B. Braun Melsungen AG, Melsungen, Germany), Remifentanil (0.3 mg/kg/h, GlaxoSmithKline / Aspen, Brentford, UK), and Dexmedetomidine (0.4 µg/kg/h, Orion Pharma GmbH, Hamburg, Germany).

After general anesthesia induction, a central venous access (Certofix, B. Braun Melsungen, Melsungen, Germany) was inserted under ultrasound guidance into the right internal jugular vein for the application of vasoactive substances. A sheath (Arrow Percutaneous Sheath Introducer Set, Teleflex Incorporated, Wayne, PA, USA) was inserted into the same vessel, also under ultrasound guidance. A urinary catheter (Actreen, B. Braun Melsungen, Melsungen, Germany) was inserted. Afterwards, the patients were transferred into the operating room. All syringe pumps were adjusted to guarantee smooth running of the medication, the patients were securely positioned to avoid pressure damage, and the heating blanket was activated to maintain body temperature. The depth of anesthesia was measured using a single-channel electroencephalogram recording from both hemispheres and was kept within the manufacturer’s recommendation. After surgery, patients were transferred to the intensive care unit under general anesthesia for extubation and postoperative care.

### 2.4. Postoperative Evaluation of Delirium and Stroke

Patients were assessed by a well-trained team with the CAM-ICU; this was completed on days one, two, and three after surgery. Patients were awake during the tests. It is possible that different nurses took the exams at different timepoints. All assessors had access to the results that were taken previously, as these were recorded in the patients’ records that were accessible anytime to all medical staff. All assessing nurses belonged to the same well-trained interprofessional team. The screening with the CAM-ICU was carried out according to the protocol described by Boettger et al. [28] and was considered positive as outlined in previous publications [7,29].

The occurrence of stroke was assessed via cranial CT scan. A non-contrast CCT was performed to exclude hemorrhagic stroke, followed by CT angiography and CT perfusion to identify a possible ischemic stroke.

### 2.5. Endpoints and Statistical Analysis

The primary composite endpoint was the occurrence of postoperative delirium on days 1–3. No a priori sample size calculation was performed due to the observational nature of this pilot study. Statistical analysis was conducted with R (R version 4.1.2). Continuous variables were summarized as the median [minimum-maximum] and compared using the Mann–Whitney U test. Categorical variables were analyzed using Fisher’s exact test or the chi-square test as appropriate. Correlations between ordinal variables were assessed using Spearman’s rank correlation coefficient, with a significance level of *p* < 0.05. In addition to univariate analyses, a multivariable logistic regression model was constructed to assess the association between preoperative transcranial Doppler findings and postoperative delirium. Due to the limited number of outcome events (*n* = 14), a parsimonious model including age and sex as covariates was specified to reduce the risk of overfitting. Given the presence of quasi-complete separation (no cases of postoperative delirium in patients with normal TCD findings), Firth penalized logistic regression was applied to reduce small-sample bias and allow stable estimation of regression coefficients.

All graphs were computed using ggplot2 in R (version 4.1.2) [30].

## 3. Results

**Figure 1:**
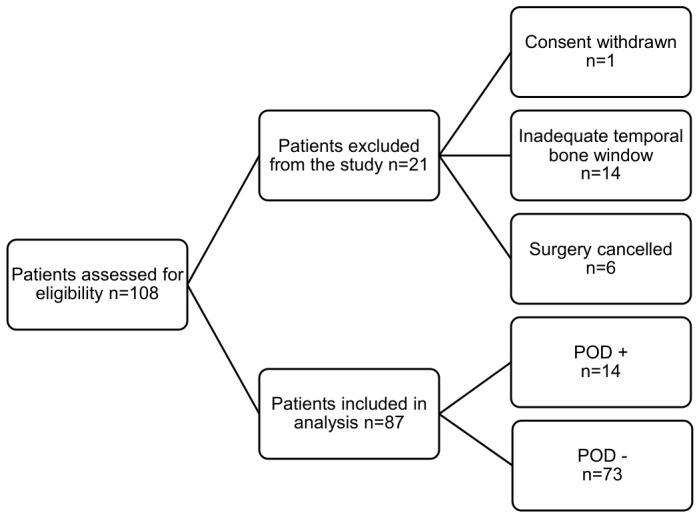
Study flow diagram.

From 108 enrolled patients, 21 had to be excluded from the study: 14 had an inadequate temporal bone window to perform the TCD; for six patients, the surgery was cancelled; and one patient withdrew their consent after surgery. Thus, 87 patients were eventually included in this study. A flow diagram summarizing patient screening, enrollment, exclusions, and final inclusion in the analysis is presented in Figure 1.

Demographic data and perioperative characteristics are presented in Table 1. Patients who developed postoperative delirium showed numerically longer aortic cross-clamp times (112 vs. 100 min, *p* = 0.44), cardiopulmonary bypass durations (160 vs. 146 min, *p* = 0.6), and extubation times (9.5 vs. 7.3 h, *p* = 0.27); however, none of these differences reached statistical significance. In contrast, the intensive care unit length of stay was significantly prolonged in patients with postoperative delirium (5 vs. 1.3 days, *p* = 0.004).

**Figure 2:**
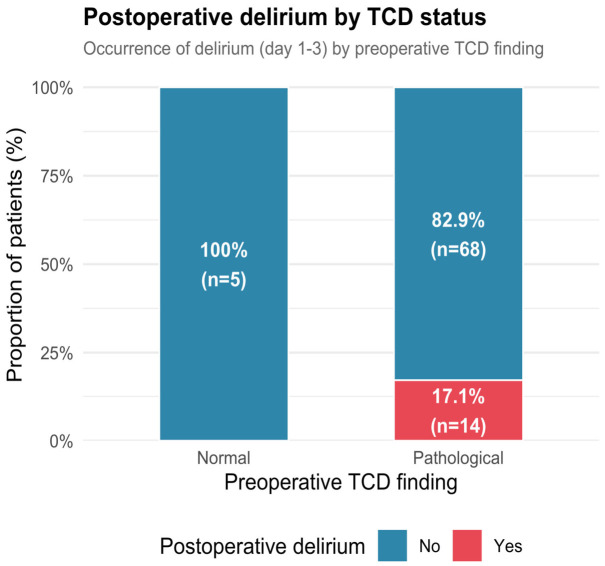
Bar plot of POD in association with preoperative TCD. POD: postoperative delirium; TCD: transcranial Doppler sonography.

Fourteen patients (16%) showed postoperative delirium, all of whom had pathological TCD. Three of all patients experienced a stroke. As the number of postoperative strokes was small, this endpoint is neglected in further discussion of the data. Those patients who had normal TCD preoperatively did not show postoperative delirium. This is shown in Figure 2.

Among the patients included in this study—all of whom had a normal carotid Doppler—82 (94%) had a pathological transcranial Doppler.

The distribution of the individual pathological TCD criteria is shown in Table 2. Reduced cerebrovascular reserve capacity (BHI < 0.69) and impaired autoregulatory response (THRR < 1.1) were the most frequently observed abnormalities, whereas a decrease in MFV of >50% following carotid compression occurred less frequently. Exploratory descriptive analyses of the individual TCD criteria demonstrated similar incidences of postoperative delirium across the different abnormalities. Because of the limited number of postoperative delirium events and the overlap between pathological TCD findings, no formal comparison of the predictive performance of the individual TCD parameters was performed.

In multivariable Firth logistic regression analysis adjusted for age and sex, pathological preoperative transcranial Doppler findings remained strongly associated with postoperative delirium. Age and sex were not independently associated with postoperative delirium in the adjusted mode. Due to the small number of events and the near-complete separation of outcomes by TCD status, the model was interpreted as a sensitivity analysis supporting the results of the univariate comparisons.

An exploratory subgroup analysis was performed to compare the incidence of postoperative delirium among patients undergoing different types of cardiac surgery. Postoperative delirium occurred in seven of 38 patients (18.4%) undergoing single-valve surgery, five of 26 patients (19.2%) undergoing multi-valve surgery, and two of 23 patients (8.7%) undergoing combined procedures. No statistically significant association between the surgical procedure type and postoperative delirium was observed (Χ^2^ test, *p* = 0.54). These data are presented in Table 3.

## 4. Discussion

The main results of the present prospective observational study are that (I) all patients who developed postoperative delirium had pathological preoperative TCD, whereas no postoperative delirium occurred in patients with normal TCD findings, and that (II) most patients had pathological TCD despite having a normal carotid Doppler. There appears to be a strong association between impaired cerebral hemodynamics and the development of postoperative delirium.

The finding that all patients who developed postoperative delirium had pathological TCD findings should be interpreted with caution. Although this observation suggests a possible association between impaired cerebral hemodynamics and postoperative neurological vulnerability, the prevalence of pathological TCD examinations in the present cohort was very high (94%). Consequently, the discriminatory capacity of the composite TCD assessment appears limited, as only a small number of patients exhibited entirely normal findings. The resulting low specificity restricts conclusions regarding the utility of TCD as a standalone predictive tool for postoperative delirium. The present findings should be interpreted not as evidence of strong predictive performance but rather as support for the hypothesis that impaired cerebrovascular function may be associated with increased susceptibility to postoperative delirium.

It is clinically relevant that normal carotid ultrasound does not necessarily imply preserved intracranial hemodynamics or intact cerebrovascular reserve. Age-related microvascular changes, endothelial dysfunction, arterial stiffness, and small-vessel disease may impair cerebrovascular reserve and autoregulatory capacity even in the absence of overt structural vascular pathology. The high prevalence of pathological TCD findings observed in the present cohort may also reflect generalized vascular aging rather than focal cerebrovascular disease. This may explain why abnormalities in functional hemodynamic parameters were frequently observed despite normal extracranial carotid ultrasound findings. Thus, TCD may provide complementary information regarding cerebral physiology beyond structural imaging.

TCD measures flow velocity rather than absolute cerebral blood flow. Although velocity is generally proportional to flow under stable vessel diameter conditions, dynamic changes during cardiopulmonary bypass, hemodilution, and variations in carbon dioxide tension may alter this relationship. A single preoperative measurement at rest may therefore not adequately reflect the hemodynamic vulnerability of the brain during surgery.

TCD is attractive as a non-invasive bedside tool that directly assesses intracranial hemodynamics. The development of self-adjusting bilateral devices overcomes previous logistical barriers requiring a dedicated examiner and may facilitate broader implementation. A single preoperative assessment may be insufficient for risk stratification. Instead, TCD might prove more valuable as an intraoperative monitoring tool to guide individualized blood pressure management and ensure adequate cerebral perfusion during cardiopulmonary bypass. Integration with autoregulation monitoring algorithms and continuous hemodynamic data could allow the identification of patient-specific lower limits of autoregulation and potentially reduce cerebral hypoperfusion.

Additionally, the observed discrepancy between extracranial and intracranial hemodynamics supports further exploration of functional cerebral assessment in elderly cardiac surgery patients. Long-term cognitive outcomes, rather than early delirium alone, may be more closely linked to chronic impairment of cerebrovascular reserve.

Perioperative variables traditionally associated with adverse neurological outcomes were also evaluated. Neither the aortic cross-clamp time nor the cardiopulmonary bypass duration differed significantly between patients with and without postoperative delirium. Likewise, extubation times were comparable between the groups. These findings suggest that the observed association between pathological preoperative transcranial Doppler findings and postoperative delirium was not primarily driven by differences in operative duration or immediate postoperative recovery. In contrast, patients who developed postoperative delirium required a significantly longer stay in the intensive care unit. This observation is consistent with previous studies demonstrating increased postoperative resource utilization and prolonged recovery in patients with delirium [6,9,12].

The study population consisted exclusively of patients aged 70 years or older undergoing cardiac surgery with cardiopulmonary bypass, representing a highly selected high-risk group with a substantial burden of subclinical cerebrovascular pathology. In such patients, functional hemodynamic impairment may be far more prevalent than structural carotid stenosis, which is consistent with our finding that all patients had normal carotid Doppler ultrasound examinations despite frequent TCD abnormalities.

An exploratory subgroup analysis comparing different surgical procedures did not demonstrate a significant association between the type of cardiac surgery and the occurrence of postoperative delirium. Although numerically lower delirium rates were observed in patients undergoing combined procedures, these differences did not reach statistical significance. This finding suggests that baseline cerebral hemodynamic impairment, as assessed via preoperative transcranial Doppler ultrasonography, may be more closely related to postoperative neurological vulnerability than the specific surgical procedure itself. However, the relatively small number of patients with postoperative delirium limits the ability to detect potentially relevant procedure-specific differences.

To further evaluate the robustness of the observed association between cerebral hemodynamic impairment and postoperative delirium, a multivariable sensitivity analysis was performed. After adjustment for age and sex, pathological preoperative transcranial Doppler findings remained strongly associated with postoperative delirium. However, interpretation of this analysis is limited by the small number of outcome events and the presence of quasi-complete separation, as no cases of delirium occurred in patients with normal TCD findings. Consequently, although the adjusted analysis supports the univariate findings, it does not allow for definitive conclusions regarding independent predictive value. Residual confounding by established risk factors for postoperative delirium, including frailty, preoperative cognitive impairment, intraoperative hypotension, transfusion requirements, and postoperative complications, cannot be excluded. Larger studies with sufficient event numbers are required to permit comprehensive multivariable adjustment and validation of the observed association.

Our findings are in line with emerging evidence suggesting that impaired cerebrovascular reserve and altered cerebral hemodynamics may contribute to postoperative neurological complications in elderly cardiac surgery patients [20,31]. Recent studies have increasingly emphasized the importance of individualized cerebral perfusion monitoring and autoregulation-guided management strategies to improve neurological outcomes [32,33].

The composite definition of pathological TCD findings incorporated several physiological domains, including cerebrovascular reserve capacity, autoregulation, and hemispheric flow asymmetry. Exploratory analyses of the individual components did not identify a single abnormality that appeared to be disproportionately associated with postoperative delirium. However, this study was not powered to determine the relative prognostic importance of individual TCD-derived parameters.

Several limitations of the present study must be acknowledged. This was a single-center study with a relatively small sample size, limiting its statistical power and generalizability. Fourteen patients were excluded due to inadequate temporal bone windows, reflecting a known limitation of TCD in the elderly population and potentially introducing selection bias. Assessors performing CAM-ICU screening had access to previous results, which may have led to observer bias.

Moreover, only early postoperative delirium on days 1 to 3 was analyzed; late-onset delirium and long-term cognitive outcomes were not evaluated. While the early timeframe captures most early postoperative delirium cases in cardiac surgical patients, delirium may also occur later during the hospital course. Therefore, the reported incidence likely underestimates the true cumulative occurrence of postoperative delirium.

A particularly important limitation is the relatively small number of postoperative delirium events observed in the present cohort. Although a prospective study design was employed, the limited event count reduced statistical power, increased the risk of unstable effect estimates, and restricted the ability to adequately adjust for multiple potential confounders. Consequently, only a highly parsimonious multivariable model could be applied, and residual confounding could not be excluded. Furthermore, the single-center nature of the study may limit the generalizability of the findings to other cardiac surgical populations and perioperative care settings. Therefore, the present results should be interpreted as exploratory and hypothesis-generating and require confirmation in larger multicenter studies.

TCD measurements were obtained only preoperatively. Intraoperative hemodynamic variables, cerebral oxygenation, and autoregulation were not analyzed in conjunction with TCD parameters. Therefore, dynamic perioperative changes in cerebral perfusion could not be correlated with outcomes. Perioperative factors may have influenced the development of postoperative delirium in this cohort. In particular, anesthetic agents are known to affect cerebral physiology and may contribute to delirium risk through mechanisms such as altered synaptic transmission, changes in cerebral metabolism, and modulation of neuroinflammatory pathways. Although anesthetic management in the present study was highly standardized, with uniform induction and maintenance protocols applied across all patients, interindividual variability in the pharmacodynamic response, the depth of anesthesia, and perioperative hemodynamic stability cannot be completely excluded. Therefore, a residual confounding effect of anesthetic exposure on postoperative delirium cannot be ruled out.

An additional limitation affecting the external validity is the exclusion of patients with pathological carotid Doppler ultrasound findings. This criterion was applied to minimize confounding from significant extracranial carotid artery disease, which may independently influence cerebral hemodynamics and neurological outcomes. However, this approach may have introduced selection bias by excluding a subgroup of patients at potentially higher baseline cerebrovascular risk. As a result, the present cohort may not fully represent the broader population of elderly patients undergoing cardiac surgery, in whom carotid artery disease is more prevalent. Therefore, caution is required when extrapolating these findings to unselected cardiac surgical populations.

Furthermore, subgroup analyses according to the surgical procedure type were exploratory in nature and may have been underpowered because of the limited sample size and low number of postoperative delirium events.

Although the present study was not designed to evaluate treatment strategies, the identification of preoperative cerebral hemodynamic impairment using transcranial Doppler may have potential implications for perioperative management. Patients with abnormal TCD findings may represent a subgroup with reduced cerebrovascular reserve and impaired autoregulation, potentially increasing their susceptibility to cerebral hypoperfusion during cardiopulmonary bypass.

In this context, TCD-based risk stratification could, in future, support individualized hemodynamic management strategies, such as stricter avoidance of intraoperative hypotension or targeting of blood pressure ranges closer to the lower limit of cerebral autoregulation. Furthermore, patients identified as high-risk may benefit from intensified delirium-prevention pathways, including closer postoperative neurological monitoring, minimization of sedative and anticholinergic medications, early mobilization, sleep optimization, and proactive management of pain and metabolic disturbances.

However, these considerations remain speculative, as no intervention based on TCD findings was implemented in the present study.

Future research should include larger, multicenter cohorts to improve statistical power and external validity. Studies combining preoperative TCD with continuous intraoperative monitoring of cerebral autoregulation and perfusion may clarify whether individualized hemodynamic targets based on cerebral physiology can improve neurological outcomes. Additionally, long-term neurocognitive follow-up would help determine whether preoperative impairment of cerebrovascular reserve is associated with persistent cognitive decline rather than early postoperative delirium alone.

Randomized controlled trials evaluating TCD-guided blood pressure management during cardiopulmonary bypass may ultimately determine whether functional cerebral monitoring can translate into clinically meaningful benefits.

## 5. Conclusions

In conclusion, this prospective single-center pilot study suggests that pathological preoperative transcranial Doppler findings are frequently observed in elderly patients undergoing cardiac surgery with cardiopulmonary bypass and may be associated with postoperative delirium. Automated TCD assessment may provide additional information on cerebral hemodynamic status beyond conventional carotid ultrasound, highlighting a potential discrepancy between structural and functional cerebrovascular assessment.

However, given the limited sample size, small number of delirium events, and restricted ability to adjust for confounding factors, these findings should be interpreted as exploratory and hypothesis-generating. This study was not powered to establish independent predictive value or causal relationships. Larger multicenter studies are required to validate these findings and to determine whether TCD-derived parameters can improve perioperative risk stratification or support individualized management strategies.

## Figures and Tables

**Table 1:** Demographic data and perioperative characteristics of the study cohort. Age, LOS, ICU LOS, extubation time, cross-clamp time, and cardiopulmonary bypass time are presented as the mean [minimum–maximum], with all other data as the total amount (percentage of the group). LOS: length of stay; ICU: intensive care unit; CPB: cardiopulmonary bypass; TCD: transcranial Doppler sonography; POD: postoperative delirium.

Variable	Overall *n* = 87	POD − *n* = 73	POD + *n* = 14	*p*-Value
Age	76 [73–78]	75 [73–78]	76 [74–78]	0.7
Sex (male)	63 (72%)	52 (71%)	11 (79%)	0.7
LOS (days)	13 [11–18]	13 [11–16]	19 [13–24]	0.016
LOS ICU (days)	3.5 [1–6]	1.3 [1–3]	5 [1–12]	0.004
Extub. time (h)	8.6 [5–11]	7.3 [3–15]	9.5 [2–19]	0.27
x-clamp (min)	107.3 [72–137]	100 [54–158]	112 [46–189]	0.44
CPB time (min)	154.7 [97–198]	146 [70–269]	160 [62–269]	0.6
TCD pathol.	82 (94%)	68 (93%)	14 (100%)	0.6
Stroke	3 (3%)	1 (1%)	2 (14%)	0.012

**Table 2:** The distribution of individual pathological transcranial Doppler (TCD) criteria and the occurrence of postoperative delirium (POD). Patients could fulfil more than one pathological criterion; therefore, the numbers in the individual categories are not mutually exclusive. The overall category includes all patients with at least one pathological TCD criterion.

TCD Criterion	Pathological (*n*)	POD Among Pathological *n* (%)	Normal (*n*)	POD Among Normal *n* (%)
BHI < 0.69	75	12 (16)	12	2 (16.7)
THRR < 1.1	68	10 (14.7)	19	4 (21.1)
MFV decrease >50%	10	1 (14.3)	77	12(15.8)
SPV asymmetry >25%	50	6 (12)	37	8 (21.6)
Overall (≥1 criterion)	82	14 (17.1)	5	0 (0)

**Table 3:** Data after subgroup analysis according to the surgical procedure. POD: postoperative delirium.

Surgical Procedure	Total (*n*)	POD − (*n*)	POD + (*n*)	POD Incidence (%POD)
Single-valve surgery	38	31	7	18.4
Multi-valve surgery	26	21	5	19.2
Combined surgery	23	21	2	8.7
Total	87	73	14	16.1

## Data Availability

Due to local legislation and privacy regulations, data can be made available only after the signing of a collaboration agreement.

## Abbreviations

The following abbreviations are used in this manuscript:

TCD	Transcranial Doppler
POD	Postoperative delirium
SPV	Systolic peak velocity
EDV	End diastolic velocity
MFV	Mean flow velocity
PI	Pulsatility index
MCA	Mean cerebral artery
THRR	Transient hyperemic response ratio
BHI	Breath-hold index
CT	Computer tomography
CCT	Cranial computer tomography
CAM-ICU	Confusion assessment method for the intensive care unit
LOS	Length of stay
